# Establishing Competencies for a Global Health Workforce: Recommendations from the Association of Pacific Rim Universities

**DOI:** 10.5334/aogh.32

**Published:** 2019-03-26

**Authors:** Mellissa Withers, Hsien-Ho Lin, Terry Schmidt, John Paul Caesar Robles delos Trinos, Shubha Kumar

**Affiliations:** 1 University of Southern California, US; 2 National Taiwan University, TW; 3 UC Irvine, US; 4 University of the Philippines, PH

## Abstract

**Background:**

The Association of Pacific Rim Universities Global Health Program facilitates exchange of information, knowledge and experiences in global health education and research among its 50 member universities. Despite the proliferation of global health educational programs worldwide, a lack of consensus exists regarding core competencies in global health training and how these are best taught.

**Methods:**

A workshop was convened with 30 faculty, university administrators, students, and NGO workers representing both the Global North and South to gain consensus on core competencies in masters’-level global health training. The co-authors then collaborated to refine the list of competencies, categorize them into domains, and develop a plan for how academic institutions can ensure that these competencies are effectively taught.

**Findings:**

Nineteen competencies across five domains were identified: knowledge of trends and determinants of global disease patterns; cultural competency; global health governance, diplomacy and leadership; project management; and ethics and human rights. The plan for how academic institutions can best train students on these competencies outlined five key opportunities: coursework; practicums; research opportunities; mentorship; and evaluation. The plan recommended additional institutional strategies such as maximizing collaborative research opportunities, international partnerships, capacity-building grants, and use of educational technology to support these goals.

**Conclusions and Recommendations:**

While further research on the implementation of competency-based training is warranted, this work offers a step forward in advancing competency-based global health masters’ education as identified by a globally diverse group of expert stakeholders and economies. Given the challenges facing the current global health landscape, comparable competency-based training across institutions is critical to ensure the training of competent global health professionals.

## Introduction

The Association of Pacific Rim Universities (APRU) is an international, non-profit consortium of 50 research universities in the Pacific Rim, representing 16 economies,^1^ 120,000 faculty members and approximately two million students. The APRU Global Health Program (GHP), launched in 2007, provides collaborative activities in research, education and training, and service around the globe. Program members include faculty and researchers who are actively engaged in global health research, education and training.

At the annual meeting of APRU GHP in 2014, participants noted that despite the great number of global health education programs across universities, the wide variation in professional training, common gaps in training, and a general lack of consensus around defining key competencies remain at issue. Furthermore, participants also underscored the need to identify and share best practices of how competency-based training could be implemented and supported across institutions with varying resources.

In academic literature, numerous researchers and academicians have highlighted the need for the development of a set of global health competencies [1,2,3,4,5,6,7,8,9,10,11,12,13]. Despite the growing interest in the field of global health and the proliferation of global health education programs worldwide, little agreement exists on what constitutes appropriate global health competencies and training [2,3,6,8,14,15,16,17,18,19]. Most of the available literature regarding this topic comes from the United States [15] (US) or North America [13,19,20,21], and may fail to offer perspectives of institutions from other countries, especially those of low- and middle-income (LMI) economies which suffer a significant proportion of the global disease burden. Occasionally, competencies are decided by faculty without consultation of other key stakeholders such as practitioners in the workforce where students will ultimately work. In addition, existing US-based research in global health competencies has focused on medical student education but few have actually identified a set of core competencies relating specifically to public health [7,15,16,18,21,22,23,24]. Accreditation requires that schools of public health demonstrate that students meet the broad set of competencies related to public health and its domains, including biostatistics, epidemiology, health policy and administration, and health promotion. Although founded on public health principles, adequate training in global health requires additional competencies unique to global health [1]. While many individual academic institutions, including the University of Toronto, the University of Washington, University of California at San Francisco, and Emory University, and professional organizations in the US have created their own set of core competencies in global health, there is considerable variation among them with no universal standards [1,24]. Among the most comprehensive examinations of global health competencies specifically for public health programs are those published by the Association of Programs and Schools for Public Health (ASPPH) in 2014 [10] and the Consortium of Universities for Global Health (CUGH) in 2014 and 2015 [11,12,25].

As the development and expansion of global health education programs continues, a set of clearly defined core competencies in global health education is necessary to ensure that students are well-equipped for their future roles as professionals, regardless of where they were trained, as consistency can help to ensure comparable training of students across programs and nations [15,26]. The two main objectives of this work were: 1) to advance a set of core competencies in master’s-level global health education developed by a panel of inter-disciplinary global health experts from eleven different economies, including low-, middle- and high-income economies; and 2) to offer recommendations on how academic institutions can best teach these competencies within a Masters-level educational program specific to global health.

## Methods

**Figure 1:**
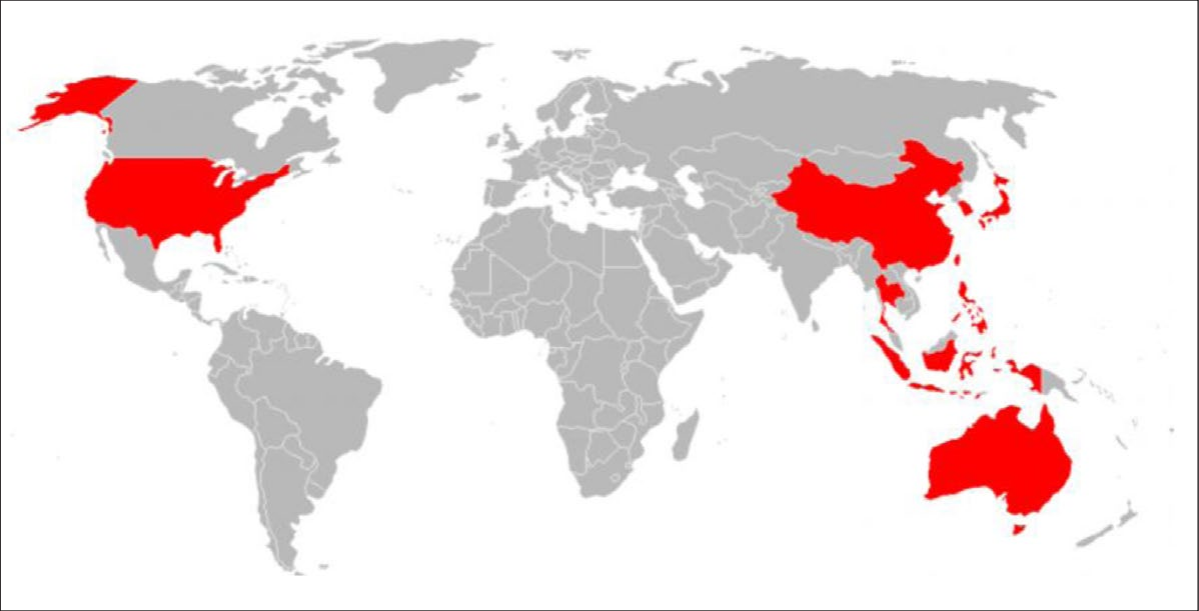
Map of Economies Represented by Workshop Participants.

This work was carried out in multiple phases. To begin, the co-authors conducted a review of the existing literature regarding core competencies in global health education and training, examining both scientific publications listed on Pubmed and Medline as well as the grey literature through Google, which included reports from various North American universities and professional associations such as the Council of Linkages Between Academia and Public Health Practice, and the Association of Schools and Programs of Public Health (ASPPH). Following this, a one-day workshop was held in conjunction with the APRU GHP’s annual conference, which was held in Taipei in fall 2014. The workshop convened an international, interdisciplinary group of thirty stakeholders who were invited because of their expertise in global health research, education and training, including university professors, administrators, students and non-governmental organizations (NGO), from 11 different economies (Australia, China, Taiwan, Hong Kong SAR, Indonesia, Japan, the Philippines, Singapore, South Korea, Thailand, and the US), as seen in Figure 1.

The workshop began with a presentation summarizing the results of the literature review and offering selected examples of models of competencies for public health developed by various organizations, such as ASPPH. Then, six small groups of five participants each were organized, with a mix of participants from at least three different economies in each group. Two co-moderators facilitated the discussions. Groups were asked to discuss two main questions: 1) what core competencies are required in global health education, focusing on masters-level students in programs related specifically to global health, but excluding physician training programs?; and 2) how do we best train students in these competencies? Through a multiple economy and stakeholder perspective, workshop participants critically reflected on the ways in which academic institutions can ensure the development of core competencies and the potential challenges that may be encountered. Participants were asked to reflect on the distinct set of challenges that educational institutions in low-, middle-, and high-income economies may face in providing comparable global health education and training. A report regarding the workshop was disseminated to all participants. The five co-authors of this paper from the Philippines, Taiwan, and the US collaborated over the next two years to refine the list of competencies, categorize them into domains, and develop recommendations for how academic institutions can best train students in these competencies.

## Results/Findings

### Domains & Competencies

At the outset, it was agreed that global health is an extension of public health, and as such core public health knowledge and skills, such as epidemiology, biostatistics, health education, policy and management, are fundamental in global health training. However, global health professionals need additional skills to perform their duties, and coursework must reflect the multidisciplinary nature of global health. The group identified five core domains specific to global health that are key additions to public health fundamentals:

1. Trends and Determinants of Global Disease Patterns.
2. Cultural Competency.
3. Global Health Governance and Diplomacy.
4. Project Management.
5. Ethics and Human Rights.

(summarized below). The group then identified 19 specific competencies within these domains (see Table 1).

**Table 1:** Domains and Competencies.


**DOMAIN: 1. Trends and Determinants of Global Disease Patterns.**	
1a.	Describe current global and country-specific disease burden and general trends for communicable and non-communicable diseases (NCDs).
1b.	Describe trends and patterns in health systems, universal health coverage, and health inequities.
1c.	Analyze how social, cultural, and economic factors influence individual or population health status.
1d.	Demonstrate how to evaluate the complex interactions between social, cultural and economic factors as they relate to health and health behaviors.
1e.	Apply key approaches to promoting health equity.
**DOMAIN: 2. Cultural Competency.**	
2a.	Analyze how social and cultural factors, such as religious beliefs, customs, and lifestyle may influence health.
2b.	Describe how colonialism, imperialism, racism and other forms of discrimination have shaped and continue to shape health and health disparities in low, middle and high-income countries.
2c.	Communicate information in a culturally-sensitive and appropriate manner according to the specific group or audience of this information such as technical experts, policy makers, lay audiences, or other relevant stakeholders.
2d.	Demonstrate verbal and written proficiency in at least one foreign language (highly recommended).
**DOMAIN: 3. Global Health Governance, Diplomacy & Leadership.**	
3a.	Describe the multidisciplinarity of global health and how population health is interdependent and interconnected.
3b.	Analyze how population health is affected by organizations and policies operating at multiple governmental levels including local, national, and international.
3c.	Analyze global governance, diplomacy and funding mechanisms of global health programs and their implications.
3d.	Analyze examples of large scale international cooperation in global health (including international trade agreements, multinational partnerships, and major governmental treaties and agreements such as the Framework Convention on Tobacco Control (FCTC) or Sustainable Development Goals (SDGs), including key stakeholders and their interests and the opportunities and challenges of such collaborations.
3e.	Describe how priority-setting in global health occurs, including the roles, influences, and complexities associated with large (often Western) donors and private sector organizations involved in determining priorities.
3f.	Identify the roles of key stakeholders including international organizations, governments, civil society and private industry and how these stakeholders and various sectors interact with each other to promote health.
3g.	Analyze past and current successes and failures in global health leadership, management and policy.
**DOMAIN: 4. Project Management.**	
4a.	Apply knowledge, skills, tools and techniques to effectively plan, implement, manage and evaluate global health projects, including financial management, human resources management, and coordination of logistics.
4b.	Design health needs assessments for local and international settings.
4c.	Analyze information, including quantitative and qualitative data and health indicators, relevant to global health projects.
4d.	Communicate, in verbal and/or written form, with team members, including international and/or local staff, to successfully plan and execute projects.
**DOMAIN: 5. Ethics and Human Rights.**	
5a.	Apply a human rights framework to health problems.
5b.	Apply ethical principles and practices in the conduct of global health research and programming.
5c.	Demonstrate familiarity with historical examples of ethics and human rights violations, and major international documents and/or treaties related to ethics and/or human rights and their applications in global health.
5d.	Identify which populations are most vulnerable to exploitation and risk of being participants in unethical research.
5e.	Demonstrate awareness of the responsibility to ensure the engagement of the community in designing, implementing, and evaluating global health programs.
5f.	Identify ways to minimize power differentials in one’s work.


#### 1. Trends and Determinants of Global Disease Patterns

Students should be able to describe the current global and country-specific disease burden and general trends for both communicable and non-communicable diseases (NCDs), as well as health systems, universal health coverage, and health inequities. In addition, an understanding of how social, cultural, and economic factors influence individuals’ and populations’ health status will help contextualize global health challenges and give students the ability to evaluate the complex interactions between these factors as they relate to health and health behaviors. Finally, students should be able to describe the importance of promoting health equity and key approaches to do so.

#### 2. Cultural Competency

Global Health educational programs can prepare students to work among populations of different cultures and learn to be sensitive to cultural differences. Students need awareness of and sensitivity to cultural diversity, and to know how to address diversity in global health policy, research and practice. Understanding how social and cultural factors may influence health, such as religion, customs, lifestyles, is central to work effectively in global health. It is also critical that they grasp how colonialism, imperialism, and racism have shaped, and continue to shape, health. During their academic careers, students should be provided opportunities to develop as global citizens through activities that help cultivate humility, maturity, and self-awareness, such as working in international teams or with vulnerable populations and spending time in resource-constrained settings. Periodic engagement in self-reflection of their own work is also invaluable in helping students develop as global citizens.

Students should demonstrate the ability to communicate effectively in a culturally sensitive manner and in a variety of international settings with a variety of audiences, including technical experts, policymakers, lay audiences and other stakeholders, both verbally and through written technical reports. They should be familiar with approaches for locating and disseminating global health data and information, which will also likely be required in their future careers. Communication barriers resulting from the inability to use the local language can present major challenges in global health practice. Foreign language training can be instrumental in being accepted into a community in another country and gaining a better understanding of a culture; therefore proficiency in at least one foreign language is recommended.

#### 3. Global Health Governance, Diplomacy and Leadership

In an increasingly globalized world, the interdependence and interconnectedness of the global population shapes the global burden of disease. Students should be able to describe how health is affected by organizations and policies at multiple governmental levels- including local, national and global. A basic understanding of global governance and diplomacy, as well as funding mechanisms for global health programs, trade agreements and the importance of large-scale global cooperation in global health is paramount to understanding global health systems. Students should also be prepared to discuss how global health priorities are determined, as well as analyze the large influence that western donors (i.e., the Bill and Melinda Gates Foundation) and the private sector have in setting global health priorities and the consequences, positive and negative, intended and unintended, of such. Knowledge of the many roles and interests of international organizations, governments, civil society, and industry and how sectors and stakeholders interact with each other in promoting health is also a crucial component of this domain. Awareness of large-scale multinational partnerships and major governmental treaties and agreements, such as the Framework Convention on Tobacco Control (FCTC) and the Sustainable Development Goals (SGDs), is considered fundamental. Finally, analyzing past and current successes and failures in global health leadership, management and policy can provide important lessons for future global health work.

#### 4. Project Management

The success of global health programs often requires proficiencies in project management and teamwork, and often at a distance. The successful implementation of a project depends on the capacity of teamwork, including the development of team leaders. It also requires collaboration with people on the ground from other agencies. Students should have opportunities to develop their own leadership skills as part of their education and training. For example, understanding the basic skills needed in project management, including finance, human resources, quality control, and local logistics, and familiarization with the design and implementation of needs assessments in international public health settings will likely be relevant for their future careers. Students should be able to locate, understand, and interpret quantitative and qualitative data and information on health indicators. Ideally, students should be able to conduct formative research and design proposals through the design and application of analytical tools for management and evaluation of global health programs.

#### 5. Ethics and Human Rights

In order for health and biomedical research to be ethically conducted, students must have an understanding of public health ethics and their application in global health research and programs. The principle that health is a human right should be emphasized and students should be trained to apply a human rights framework to health problems. Students should be familiar with historical examples of ethics and human rights violations, and be able to identify some of the major international documents or treaties relating to ethics and/or human rights. This includes an understanding of the three basic principles of ethical conduct in research with human subjects and how these apply in global health. They should understand which populations are most vulnerable to exploitation and risk of being participants in unethical research. Experiences within and outside of the classroom should promote professional conduct. Awareness of their own responsibility to ensure the engagement of the community in designing, implementing, and evaluating global health programs and how to minimize power differentials in their own work can help prepare students for their future careers. This includes careful consideration of the benefits and potential harms of short-term practicum experiences abroad and students who participate in such programs should be able to identify the importance of mutual benefits of such experiences.

### How to Train Students in These Competencies

The five recommendations for how academic institutions can best train students in these competencies include:

1. Coursework.
2. Practicums.
3. Research Opportunities.
4. Mentorship.
5. Evaluation.

#### 1. Coursework

Core competencies should be incorporated into coursework and curricula should be developed with these competencies in mind. Students should be aware of the expected core competencies and which courses and opportunities will help them meet each competency. Coursework should contain the theoretical basis for public health, as well as reflect the practical application of theoretical concepts.

Case studies are valuable learning tools and should be incorporated into the curriculum to provide real-world examples of global health challenges and solutions. For example, the case study entitled “loveLife: Preventing HIV among South African youth” (available online through the Harvard Global Health Delivery case collection) can be incorporated into a relevant course to have students practice the competency “analyze how population health is affected by organizations and policies operating at multiple governmental levels including local, national, and international.” Such exercises also help illustrate the complex decisions that are often involved in global health research and practice, and that many solutions and challenges are not straightforward but are often culturally-determined and contextually-driven. The field experience of prior student practicums can also be presented as case studies to facilitate understanding of the core competencies and provide a foundation for students’ own experiential learning.

Institutions, especially those in resource-constrained settings, may face challenges in designing curricula and programs that will ensure that students can achieve these competencies. The difficulty of providing global health training through a limited number of courses, which is the reality at many universities, was recognized. Institutions often have limited faculty expertise or ability to teach global health courses and may have difficulties in identifying or attracting faculty from various disciplines and/or diverse cultures, which may also reduce the range of opportunities for student research projects and practicums.

One approach that can transcend limited resources is for institutions to take advantage of available technology to create educational learning environments where students can engage with students and faculty from other universities along with global health practitioners throughout the world. Both asynchronous and synchronous techniques can provide value, including electronic discussion boards, group projects, and live videoconference sessions for real-time interaction and lively debate. Distance education courses hosted at one institution can also be opened to other institutions. An example of this is the distance education course on global health ethics that the GHP organizes, in which multiple member universities across the region participate simultaneously. In addition, a variety of free online courses that exist, such as those developed by CUGH or Johns Hopkins University. One good resource for cultural competency development is the Harvard Business School’s Global Collaboration online training. Furthermore, grant opportunities, which include assisting in the development of curricula or implementation of training activities, are often available in high-income countries. Academic institutions with limited resources can seek out partnerships with these institutions to help build capacity in global health education and training.

Distance education courses can also prepare students to learn how to work in virtual teams with colleagues in different parts of the world, since global health practice is typically conducted in team settings with members from various backgrounds and cultural contexts. Students can develop and hone skills in research, ethics, and cultural competency with these experiences. As advances in technology continue to transform, education in this realm should take advantage of the benefits technologies can provide [25].

#### 2. Practica

It was also recognized that many core competencies are best acquired outside the classroom. Therefore, a more interactive approach is preferred over a purely didactic one. Experiential learning through practicums should be considered an essential component of global health education and training [27,28]. Problem-based learning that helps students to develop required competencies should be emphasized in both the coursework and practicum experiences [28]. It is through such opportunities that students gain hands-on experience and become more competitive in the field. Practicums promote student career development by providing opportunities to expand their professional networks. Hosting international practicum students can also be a valuable experience for students from the host institution, as they will gain exposure to different cultures and perspectives [28].

However, practicums should offer more than a very short field exposure; instead they should provide the ability to directly observe the cultural context that influences health, to interact with locals, and to apply what they have learned in the classroom in real-world settings. For example, practica could be used to train students in competencies such as “analyze how social and cultural factors, such as religious beliefs, customs, and lifestyle may influence health” or “communicate information in a culturally-sensitive and appropriate manner according to the specific group or audience of this information such as technical experts, policy makers, lay audiences, or other relevant stakeholders.” A multitude of ethical and practical issues should be considered in terms of global health practica, such as concerns about sustainability of programs, overburdening the host institution, lack of adequate mentorship in the host country, limited skills of students, and lack of mutual benefits for host and sending institutions, as pointed out by Crump and Sugarman and others [29,30,31,32,33,34]. Students, faculty, and mentors should work together to establish the anticipated goals of the practicum. To adequately prepare students for global practica, institutions should hold pre-trip orientations to provide detailed information about the local cultural context and the existing health-related literature relating to that country. Prior students can be invited to these orientations to share their own experiences. Students should also have some training in ethics, including community-based participatory research where applicable. During the practicum, students should be required to critically reflect on their experiences and communicate regularly with mentors. Institutions must provide supportive and consistent mentorship to students [30,31]. Throughout the practicum, students should record their daily or weekly work in a logbook or on a blog, which can later be reviewed and evaluated by mentors. They may also be encouraged to develop electronic portfolios which showcase their work during the practicum that they could later use to attract potential employers reflect on their experiences, and highlight the benefits of the practicum for the local community.

Funding to support faculty and student travel for global health practicums is often limited and faculty and students may have to compete for economic resources as universities continue to struggle with budget constraints. Such constraints often make international practicums cost-prohibitive and thus unavailable to many students. However, practicums are an integral part of global health training and academic institutions should consider these experiences to be a priority, as they have numerous benefits. For students, such benefits may include the acquisition of new knowledge and skills, including language proficiency, empathy, and cultural competency, increased awareness regarding global health inequities and determinants of health in contexts other than their own, and greater self-confidence [28,35,36,37,38,39,40]. Students may also view practicums as a way to become more competitive in their future academic or professional careers [34,36,37,41]. Offering practicum opportunities can also be advantageous to the academic institution in terms of recruitment of students as well as meeting objectives to provide global education that many institutions have as part of their mission [30,34]. Institutions that host students should also engage with the sending institutions to establish exchange opportunities to help send their own students abroad. Institutions in high-income countries that send students abroad to host institutions in low-income countries are encouraged to find creative ways to help identify and provide resources for bi-directional exchanges of students, so that students from LMI economies are hosted at their institutions. A bi-directional exchange system can help promote more, equitable reciprocal relationships among partners. It may be possible for institutions to build these fees into training grants. Networks or groups of universities can work together to create shared projects if they are working in the same host country. As Withers et al. (2017) pointed out, more coordination could help avoid duplication of efforts by different institutions working in the same location and can reduce the burden to the host institution. For example, within APRU, multiple universities send students during the summer to work together in one location with one faculty member overseeing the group [38].

#### 3. Research Opportunities

Given that many of the core competencies in global health are best mastered in practice, research opportunities that include interaction with public health practitioners and engagement with communities are very beneficial for students. At the graduate level, global health education can be enhanced through working on faculty-led research projects in other countries such as joint collaborative research between students’ academic institutions and other community-based organizations, for example. Such opportunities could help train students in mastering competencies such as “apply ethical principles and practices in the conduct of global health research and programming” or “analyze information, including quantitative and qualitative data and health indicators, relevant to global health projects.” Long-term collaborative research requires resources and commitment, but can be mutually beneficial to both the researchers and the community by tackling major public health issues, building capacity among community-based organizations, and contributing to community empowerment. However, these projects should be developed with significant input from the community and should build upon previous work in order to be sustainable. Other opportunities for students to work with faculty on research and policy objectives through research internships or part-time research positions can be useful in terms of allowing students to produce products that demonstrate the mastery of the required competencies [28].

Global health programs may also promote or evaluate themselves on the number of faculty and students working on projects abroad, the various countries they are working in, and the array of projects that are being conducted. Offering such opportunities for students should be considered a requisite. Given the interdisciplinary nature of global health, schools and programs can work together to offer a range of research opportunities across many disciplines.

#### 4. Mentorship

High-quality mentorship is crucial for graduate students to be able to adequately develop core competencies in global health. While a faculty member is one type of mentor, students can also learn different aspects of global health from academic and field mentors. Educational programs should seek to provide students with both types of mentorship. We acknowledged that mentorship can take a considerable amount of time and is often not evaluated as part of faculty reviews and little financial compensation usually is given for mentorship activities. Training and incentives should be given to faculty to facilitate and enhance the interaction between mentors and students, and evaluation of faculty should include mentorship activities in order to acknowledge and reward good mentorship. In addition, student-to-student mentorship can be valuable.

#### 5. Evaluation

It is not enough to simply offer such programs; a standardized system for evaluating their quality should be developed. Establishing some consensus on core competencies in global health and how to evaluate mastery of them could help to ensure comparable training of students and faculty across programs and nations [15,26,35]. A major challenge includes how to effectively evaluate and measure the success of students in meeting the core competencies in global health education and training programs. The achievement of core competencies should be evaluated in many ways, using both qualitative and quantitative methods. While the basic knowledge of global health can be examined through standard student evaluation methods such as major exams or assignments in individual courses and/or via a final comprehensive exam or thesis, competencies should also be measured separately. Students should perform self-assessments by completing a checklist or audit of core competencies at the beginning and end of their degree programs. In addition, students should partake in peer evaluations to assess their peers’ interpersonal dynamics, cultural competency, and other such skills. Students can also complete self-assessments and field mentors can be called upon to evaluate the students’ demonstration of competencies, progress and success in meeting practicum objectives. Finally, academic institutions should also track the career development of alumni; by examining which core competencies are used in alumni positions, academic institutions can be confident that the core competencies are useful, appropriate and relevant in public health practice. Governments and private institutions are also encouraged to define criteria for accreditation and certification of global health professionals [42,43,44].

## Discussion

The importance of expanding global health education and training is unquestioned. However, programs should be built around the achievement of core competencies. This paper presented the results of efforts from 30 participants working in global health from 11 economies in the Global North and South who shared their perspectives on core competencies of global health for graduate students. Although this contribution comes from institutions in the Pacific Rim, the core competencies are seen as applicable to any region and institution. This is one of the few published descriptions of efforts to establish a consensus from such a diverse perspective. Twelve key conclusions of this paper are shown in Table 2 below.

**Table 2:** Key Conclusions of This Study.


1.	Global health training requires additional competencies in addition to the basic public health competencies.
2.	The use of a competency-based model for global health programs can help to guide academic institutions on how to best prepare the next generation of global health leaders.
3.	Standardizing the minimum requirements for competency in global health across institutions and nations can ensure adequate training of future global health leaders, no matter where they work.
4.	Core competencies should be incorporated into coursework and curricula should be developed with these competencies in mind.
5.	Five mechanisms through which global health programs can best train students on these competencies are: coursework; practicums; research opportunities; mentorship; and evaluation.
6.	Case studies are valuable learning tools and should be incorporated into the curriculum to provide real-world examples of global health challenges and solutions.
7.	Institutions should take advantage of available technology to create educational learning environments where students can engage with students and faculty from other universities and with global health practitioners throughout the world.
8.	Practicums are indispensable components of global health educational programs at the master’s-level.
9.	Since many of the core competencies in global health are best mastered in practice, research opportunities that include interaction with public health practitioners and engagement with communities is very beneficial for students.
10.	High-quality mentorship by faculty mentors, field mentors, and fellow students should be considered an indispensable component of all global health educational and training programs.
11.	Establishing some consensus on core competencies in global health and how to evaluate mastery of them could help to ensure comparable training of students across programs and nations.
12.	Governments and private institutions are also encouraged to define criteria for accreditation and certification of global health professionals.


Our efforts were focused on providing general master’s-level competencies for global health that can be applied across academic institutions, regardless of their geographical location. Our group delineated five core domains in global health, in addition to the standard public health core competencies with 19 specific competencies.

As indicated by other scholars, global health core competencies should reflect the interdisciplinary nature of the field [24,47]. In comparing our core competencies with the literature, our set of core competencies overlapped in many areas with what has been developed by others. For example, in their literature review of 13 articles, largely coming from North America and Europe, Sawleshwarkar & Negin (2017) identified 11 domains relating to postgraduate global health education that were most common, which were grouped into three broad areas: 1) burden of disease and determinants of health; 2) core skills, such as policy development and program management; and 3) soft skills, such communication and collaboration [21]. The Association of Schools and Programs for Public Health (ASPPH) recommends 38 competencies within seven domains for master’s-level education in global health [10]. Finally, the CUGH global health competency framework incorporates four levels of global health competence and inclusive sets of interprofessional competencies for global health practitioners. The framework outlines eight general global health domains with 13 competencies. The eight domains for global citizen are: global burden of disease; globalization of health and health care; social and environmental determinants of health; collaboration, partnering, and communication; ethics; professional practice; health equity and social justice; and sociocultural and political awareness. In addition to the eight domains listed for the global citizen level, 26 additional competencies for program-oriented basic operational level are included in three broad domains capacity strengthening, program management, and strategic analysis. Each competency is categorized as knowledge, an attitude and/or a skill [11,12].

While there was much overlap in the competencies recommended by other groups and ours, one unique element of ours is that we highly recommend foreign language proficiency. In addition, we outline a set of five specific recommendations for academic institutions to ensure that students develop these competencies during their educational programs. Another distinction of our recommendations is the global health practicum, which we deemed critical in order to promote the acquisition of these competencies. Opportunities for students to develop their own leadership skills, as well as exposure to ethics training should be considered necessary components of global health education. Appropriate graduate-level training in global health requires both adequate and appropriate related coursework, as well as a formal practicum, ideally in a foreign country. Such experiences allow students to integrate knowledge acquired in a classroom into a real-world setting. Skills such as cultural competency can be best developed during such experiences; practicums can be instrumental in terms of building global citizens and civic engagement [44]. However, we noted that pre-departure trainings are crucial to adequately prepare students for global health practicums, as recommended in other studies [32,34,45,46,47].

In addition, our paper emphasizes that programs should incorporate 21st-century technology [48]. As others have highlighted, the ease of communication and innovations in information technology offers new opportunities in terms of distance education that can connect students from numerous universities [46,47,48]. While such courses can be logistically complicated and difficult to manage, they can also be invaluable in terms of building capacity and sharing knowledge amongst participants from the Global North and South. They can also help foster teamwork and a sense of a global community.

As highlighted by Macfarlane (2008), offering global health educational programs is often seen as a way to increase prestige of an academic institution in high-income settings. However, academic institutions in LMI economies should also consider developing global health educational and training programs that can build partnerships across institutions and economies. These opportunities also ensure that they have significant input in developing capacity of future global health leaders who will be poised to address health disparities and future global health challenges [5,39].

## Conclusion

Academic institutions play a major role in the preparation of the global health workforce and the use of a competency-based model for educational and training opportunities can help guide academic institutions on how to best prepare the next generation of global health professionals. In order to adequately prepare students for the unique challenges of working in global health, specific competencies in global health must be met. Along with other scholars, we acknowledge some of the major challenges that academic institutions may face in the implementation of such competencies and we offer some possible strategies to address these challenges. It is our hope that this paper will serve as a resource for academic institutions in the process of creating new or revising existing global health programs.

## Footnotes

APRU’s policy is to use the word economies instead of “countries” in order to include Taiwan and Hong Kong SAR.
